# Multidrug-resistant tuberculosis outbreak in an Italian prison: tolerance of pyrazinamide plus levofloxacin prophylaxis and serial interferon gamma release assays

**DOI:** 10.1016/j.nmni.2016.03.010

**Published:** 2016-04-07

**Authors:** A. Bedini, E. Garlassi, C. Stentarelli, S. Petrella, M. Meacci, B. Meccugni, M. Meschiari, E. Franceschini, S. Cerri, A. Brasacchio, F. Rumpianesi, L. Richeldi, C. Mussini

**Affiliations:** 1) Clinic of Infectious Diseases, Azienda Ospedaliero-Universitaria, Policlinico of Modena, Modena, Italy; 2) Medical Department, Sant'Anna Penitentiary, Modena, Italy; 3) Service of Microbiology, Azienda Ospedaliero-Universitaria, Policlinico of Modena, Modena, Italy; 4) Clinic of Lung Diseases, University of Modena, Modena, Italy; 5) Clinic of Infectious Diseases, University of Modena and Reggio Emilia, Modena, Italy; 6) Department of Infectious Diseases, Ospedale Santa Maria Nuova, Reggio Emilia, Italy; 7) University of Southampton, Southampton, UK

**Keywords:** Hepatotoxicity, latent tuberculosis infection, levofloxacin, multidrug-resistant tuberculosis, pyrazinamide, QuantiFERON-TB Gold In-Tube

## Abstract

The optimal treatment for latent tuberculosis infection (LTBI) in subjects exposed to multidrug-resistant (MDR) tuberculosis (TB) remains unclear, and the change in response of the QuantiFERON-TB Gold In-Tube (QTB-IT) test during and after treatment is unknown. Between May 2010 and August 2010, 39 prisoners at the ‘Casa Circondariale’ of Modena, Italy, were exposed to a patient with active pulmonary MDR TB. All contacts were tested with the tuberculin skin test and QTB-IT. Upon exclusion of active TB, subjects positive to both tests were offered 6 months' treatment with pyrazinamide (PZA) and levofloxacin (LVX). QTB-IT testing was repeated at 3 and 6 months after initial testing in all subjects who were offered LTBI treatment. Seventeen (43.5%) of 39 subjects tested positive to both tuberculin skin test and QTB-IT test, and 12 (70.5%) agreed to receive therapy with PZA and LVX at standard doses. Only five (41.6%) of 12 subjects completed 6 months' treatment. Reasons for discontinuation were asymptomatic hepatitis, gastritis and diarrhoea. The QTB-IT values decreased in all subjects who completed the treatment, in two (33%) of six of those who received treatment for less than 3 months and in one (50%) of two patients who discontinued therapy after 3 months. The QTB-IT test results never turned negative. Despite the small number of subjects, the study confirmed that PZA plus LVX is a poorly tolerated option for MDR LTBI treatment. We observed a large degree of variation in the results of the QTB-IT test results among participants. The study confirmed that the interferon gamma release assay is not a reliable tool for monitoring the treatment of MDR LTBI in clinical practice.

## Introduction

Multidrug-resistant (MDR) tuberculosis (TB) is defined as *Mycobacterium tuberculosis* that is resistant at least to isoniazid (INI) and rifampicin (RIF). Extensively drug-resistant (XDR) TB is defined as *M. tuberculosis* resistant to INI, RIF, any fluoroquinolone and at least one of three injectable second-line drugs (capreomycin, kanamycin and amikacin) [1]. As the number of people with MDR TB or XDR TB increases, so does the number of their contacts, and it is precisely these contacts who need to be identified and properly managed. The management of contacts of MDR and XDR TB patients is particularly challenging, as the evidence for the best intervention is limited. In drug-susceptible TB, preventive therapy in individuals with latent TB infection (LTBI) has been shown to be effective in reducing the future risk of developing TB disease [2]. The concept is also valid for MDR and XDR TB but is limited by the current lack of availability of effective drugs against MDR and XDR TB infections, with an unacceptable adverse event (AE) profiles in otherwise healthy individuals. During the past decades, the treatment recommended for LTBI in contacts exposed to MDR TB was pyrazinamide (PZA) combined with either ethambutol (ETB) or a fluoroquinolone [3]. These recommendations were supported by expert opinion but not by controlled trials [4]. Some reports have highlighted the potential hepatotoxicity of combined treatments of PZA plus ETB and PZA plus levofloxacin (LVX) for MDR LTBI [5], [6], inducing the World Health Organization and the international community to change the approach to the contacts of MDR TB cases, preferring a careful clinical follow-up of 2 years rather than antibiotic treatment [7].

Interferon gamma (IFN-γ) release assays (IGRAs) are important tools for LTBI diagnosis and surveillance for new TB infection [8], [9], [10]. IGRAs are *in vitro* assays based on the detection of IFN-γ production in response to early-secreted antigenic target 6 kDa protein (ESAT-6) and culture filtrate protein 10 (CFP-10). These antigens are specific to *M. tuberculosis* and are absent from all bacillus Calmette-Guérin vaccine strains and most environmental mycobacteria [11], [12]. The QuantiFERON-TB Gold In-Tube (QTB-IT; Cellestis, Valencia, CA, USA) test contains a third *M. tuberculosis*–specific antigen (TB7.7) and uses an enzyme-linked immunosorbent assay for detection of IFN-γ responses. The US Centers for Disease Control and Prevention have recommended the use of the QTB-IT test as an appropriate substitute for the tuberculin skin test (TST) in contact investigations [13]. Interpretation of serial IGRAs is challenging because of nonspecific variation, conversions and reversions. Previous studies have proposed that conversions, reversions and nonspecific variations occur with both serial IGRAs and TST [14], [15], [16], [17], [18], [19]. In addition, previous TST results may boost the subsequent IGRA responses, rendering the interpretation of serial IGRA results more difficult [20]. Until now, in LTBI subjects, most serial IGRAs were performed after INI or rifampin preventive treatment [21], [22], [23]. However, to our knowledge, there are no reports evaluating serial IGRAs after treatment for MDR LTBI with PZA plus LVX.

The aim of the present study was to evaluate the tolerance of the MDR LTBI treatment and the kinetics of QTB-IT in three groups of patients: those who concluded 6 months' treatment with PZA and LVX, those who received less than 6 months' treatment and those who refused treatment.

## Patients and Methods

One inmate of ‘Casa Circondariale S. Anna’ penitentiary (Modena, Northern Italy) was diagnosed with active pulmonary TB in August 2010. The case was diagnosed with bacteriologically confirmed pulmonary TB, and susceptibility testing of the *M. tuberculosis* strain showed resistance to RIF and INI as well as reduced susceptibility to ETB (resistance to 5.00 μg/mL, susceptibility to 7.50 μg/mL). The investigation of the outbreak was initiated by performing TST and QTB-IT tests in all individuals who had contact with the index case during the 2 months before the diagnosis. To exclude other cases of active TB, all the individuals who had positive results for both the TST and QTB-IT test underwent chest X-ray and a high-resolution computed tomographic (HRCT) scan of the chest. All patients who tested positive by TST and QTB-IT with a normal chest HRCT result were considered for 6 months' directly observed LTBI treatment regimen including PZA and LVX. Acceptance of LTBI treatment required written informed consent. The prophylactic regimen was administrated by directly observed therapy. HIV, hepatitis C virus and hepatitis B virus serology were performed before treatment; liver function testing (aspartate aminotransferase (AST) and alanine aminotransferase (ALT)) was performed at baseline, 2 weeks after the beginning of treatment and then monthly. LTBI treatment was discontinued if the increase in ALT or AST was greater than four times the upper limit of normal (ALT, 42 IU/L; AST, 42 IU/L) or if the patient experienced drug-related AEs.

The QTB-IT test was repeated after 3 and 6 months in all subjects.

### Tuberculin skin test

TST was administered using the Mantoux method with 2TU PPD RT23 (Statens Serum Institute, Copenhagen, Denmark) [3]. The induration size was measured after 48 to 72 hours by a trained medical doctor, and a 10 mm induration size was set as the cutoff value.

### QTB-IT test

All participants were tested by QTB-IT as per the manufacturer's instructions (http://www.quantiferon.com/irm/content/quantiferon-tb-gold1.aspx?RID=300). An IFN-γ response to the ESAT-6/CFP-10/TB7.7 mixture ≥0.35 IU/mL above the nil control value (and ≥25% of the nil control) was considered a positive result for the QTB-IT test. If a response to *M. tuberculosis*–specific antigens (corrected for the nil control) was <0.35 IU/mL and the response to the positive control was >0.5 IU/mL, then the response was considered negative. Indeterminate results were classified as nil-corrected IFN-γ responses <0.35 IU/mL and positive control responses <0.5 IU/mL. QTB-IT test reversion was arbitrarily defined as a change from a positive (≥0.35 IU/mL) to a negative (<0.35 IU/mL) result.

When the QTB-IT antigen-specific value was >10.0 IU/mL, we performed a 1:10 dilution of the plasma samples and we repeated the IGRA determination; finally, the QTB-IT value was multiplied by 10.

## Results

### Clinical characteristics

**Fig. 1:**
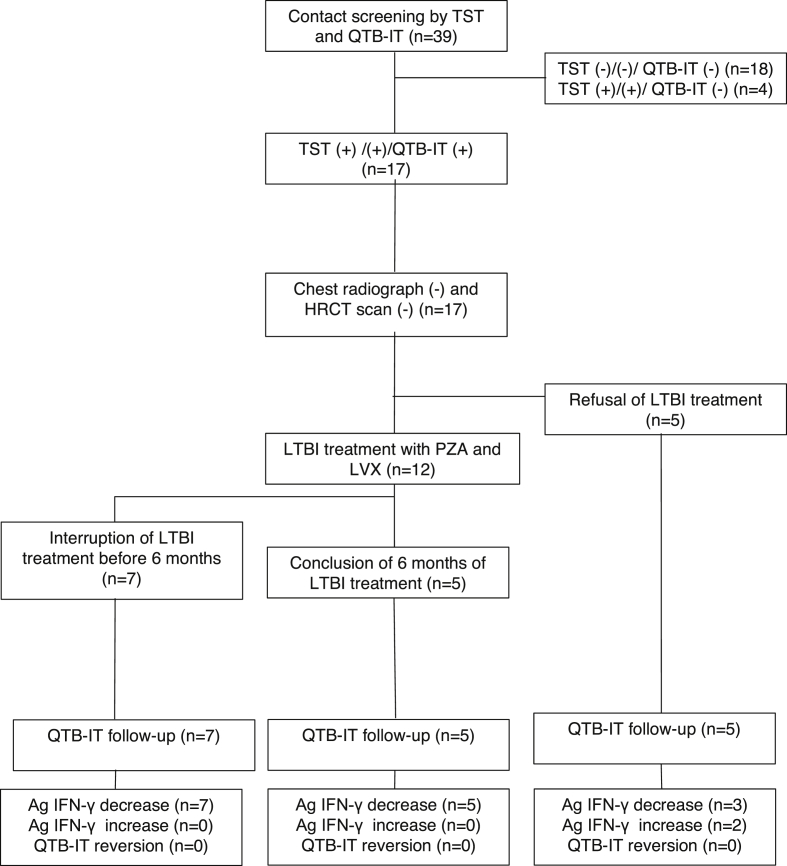
Flow diagram of contact investigation of infectious tuberculosis patients (*n* = 39). Ag IFN-γ, antigen-specific IFN-γ; LVX, levofloxacin; PZA, pyrazinamide; QTB-IT, QuantiFERON-TB Gold In-Tube; TST, tuberculin skin test.

A total of 39 subjects were screened with TST and QTB-IT test, which were positive in 58.9% and 43.5%, respectively. The 17 individuals with positive TST and positive QTB-IT test underwent chest x-ray and HRCT scan. Results of the radiologic examinations were all negative, and the 17 patients were included in the study (Fig. 1, Table 1). The subjects were all men with a mean age of 34 years (range, 21–51 years); 13 (76.5%) were foreign born and came from countries highly endemic for TB (Table 2). The LTBI treatment with PZA (20–25 mg/kg/day) and LVX (500 mg/day) was offered to all 17 individuals but was accepted by only 12 (70.5%) of them. The patients who received LTBI treatment had a mean weight of 70 kg (range, 59–95 kg), and all were negative for HIV, hepatitis C virus and hepatitis B virus infection. All the patients were smokers, and four (33.3%) of 12 subjects were regularly taking benzodiazepines or neuroleptic drugs. The median duration of LTBI treatment was 108 days (range, 27–180 days); only five patients (41.6%) completed the 6-month course of therapy. The main AE observed during the LTBI treatment was asymptomatic hepatitis (*n* = 6, 50.0%), followed by diarrhoea (*n* = 2, 16.6%), gastritis (*n* = 2, 16.6%), headache (*n* = 1, 8.3%) and arthritis (*n* = 1, 8.3%). In one patient (8.3%), an asymptomatic increase of plasma uric acid concentration occurred. The number of subjects reporting AEs and the number of therapy discontinuations due to AEs are reported in Table 3.

### Serial QTB-IT test

**Fig. 2:**
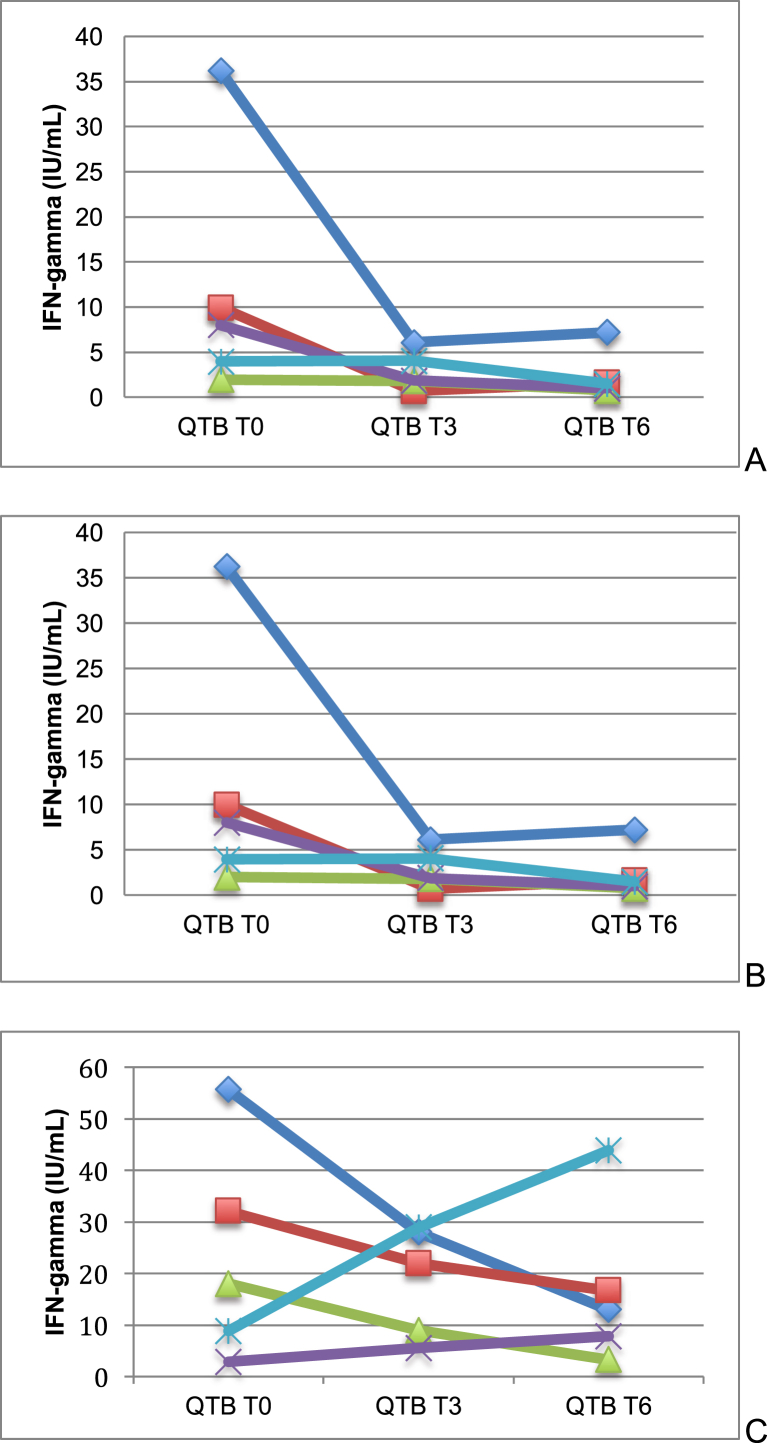
Kinetics of QTB-IT tests in patients who received 6 months' LTBI treatment with PZA and LVX (A), patients who interrupted treatment before 6 months (B) and patients who refused treatment (C). LTBI, latent tuberculosis infection; LVX, levofloxacin; PZA, pyrazinamide; QTB-IT, QuantiFERON-TB Gold In-Tube.

The QTB-IT test was repeated after 3 and 6 months in all subjects who were initially offered LTBI treatment (*n* = 17). The kinetics of QTB-IT tests is shown in Fig. 2 and Table 2. The QTB-IT test never converted to negative, but antigen-specific IFN-γ (Ag IFN-γ) levels decreased in all subjects who completed the treatment and in all patients who received <6 months' LTBI treatment. No relationship between baseline TST induration sizes and extent of decrease of QTB-IT test values was observed. The kinetics of the Ag IFN-γ levels in the five patients who refused prophylaxis showed discordant results: in three patients (60.0%) we observed an increase of the QTB-IT value, and in two (40.0%) we observed a decrease (Fig. 1). During 24 months' follow-up, none of the patients developed active pulmonary or extrapulmonary TB.

## Discussion

Although the incidence of MDR TB is low, one of the most important strategies to contain its spread would be the prevention of active MDR TB through effective LTBI treatment. Preventive therapy of patients with drug-susceptible LTBI has been proven to be effective and is included in guidelines for TB control [22]. Unfortunately, no treatment regimens for MDR LTBI have been tested in a randomized, controlled trial [24]. Therefore, the question remains of how to treat MDR LTBI, or even whether to treat it. Attamna *et al*. [25] described the incidence of MDR TB disease in close contacts (*n* = 476) of patients with pulmonary MDR TB (*n* = 78) after preventive therapy compared to the incidence in close contacts who did not receive preventive therapy. The study was performed in Israel between 1998 and 2006. Follow-up was provided for a minimum of 3 years, with a maximum of 8 years. In this study, no cases of TB occurred in either the treated or the untreated group, so therefore this study provides very limited evidence. In another study, Schaaf *et al*. [26] performed a prospective cohort study in infected (*n* = 61) and noninfected (*n* = 44) children younger than 5 years in household contact with adults with pulmonary MDR TB (*n* = 73). The study was conducted in South Africa between 1994 and 2000. All infected children and all children younger than 2 years who had received no previous treatment or preventive therapy of any kind for TB were offered preventive therapy. This study provides evidence that preventive therapy—taking into account the resistance profile of the index case—may prevent TB disease in children (under the age of 5 years) who are in contact with MDR TB patients. The risk of developing TB disease was lower in the treated group, but the risk difference was not significant, with a risk difference of 5% (95% confidence interval, −2 to 11) in favour of preventive therapy. The study found a significant risk difference of 15% (95% confidence interval, −27 to −4) between treated and untreated children when assessing confirmed and probable TB. All three culture-confirmed TB cases occurred in children not receiving preventive therapy.

In our study, we decided to treat the MDR TB contacts who were diagnosed with LTBI because they lived in a closed environment—a prison—and we considered there to be an elevated risk of transmission in case there would be a new episode of active MDR TB. Because the strain of *M. tuberculosis* isolated from the index case was resistant to RIF and INI, and because it showed reduced susceptibility to ETB, we decided to treat LTBI with a combination regimen of LVX and PZA. Limited data are available on the safety profile of this regimen when used for LTBI treatment. A systematic review assessed the AEs in anti-TB preventive therapy (drugs other than INI or RIF) in healthy individuals [27]. For LVX (six studies, eight study arms) no severe AEs were reported, and the reason for dropout was not related to AEs. For PZA, which is always used in combination with other drugs, four case series on MDR LTBI treatment are available. Combination therapy was prescribed for 6 to 12 months (PZA with ofloxacin in two studies, with ETB in one study and with LVX in another study). All these studies reported a high frequency of AEs. Treatment was discontinued in 58% to 100% of the subjects as a result of AEs that ranged from mild, such as nausea and dizziness, to serious ones requiring treatment. An increase in liver enzymes was a reason for treatment discontinuation in 25% to 58% of cases.

In our population, the combination of PZA and LVX was poorly tolerated, with a rate of treatment interruption of 58.4%. However, comparing our case cohort with that studied by Papastavros *et al*. [6] (17 individuals with suspected MDR LTBI treated with PZA and LVX), we observed a lower incidence of AEs related to the musculoskeletal system (8.3% and 82.3%, respectively), central nervous system (0 and 29.4%, respectively) and skin (0 and 29.4%, respectively), as well as a lower rate of increase in plasmatic uric acid (8.3% and 47.0%, respectively). In addition, the median time of LTBI treatment was longer in our case cohort (108 and 32 days, respectively). One possible explanation is that the prisoners may have had a higher pain threshold than a nonprison population. Another is that the prisoners had a greater adherence to prophylaxis because their therapy was directly observed, and the patients had weekly visits with the infectious diseases specialist during the treatment period. In our population, only three subjects (25.0%) did not experience any AEs. In six patients (50.0%) an increase in AST and ALT was observed, and in five of them (41.6%) the increase was fourfold greater than the upper value of the normal range and required treatment interruption. In all cases, toxic hepatitis was asymptomatic; it was diagnosed by monthly screening of liver enzymes.

To our knowledge, this is the first study to evaluate the kinetics of QTB-IT in patients receiving preventive treatment for MDR LTBI. Recent research has focussed on the use of IGRAs as a biomarker indicating treatment success [28], [29]. It has been postulated that a decrease in the magnitude of IFN-γ responses to *M. tuberculosis*–specific peptides measured by IGRA can be used as a biomarker of cure [30]. In 2013 Denkinger *et al*. [31] published a large study on the use of IGRA to monitor anti-TB treatment response in 149 patients with active TB. A large proportion of patients remained QTB-IT positive even after completing treatment. The authors also found substantial within-subject variability in sequential measurements despite all patients receiving adequate therapy and having nearly 100% adherence. They suggested that this within-subject variability might have been influenced by exogenous (i.e. incubation time, contamination with other antigens, different test operators, other analytical inconsistencies) and endogenous factors (i.e. antigen burden, cumulative TB exposure, time interval from exposure, clearance of infection, recent TST, concomitant infections, medications, other unknown factors). In 2015 Clifford *et al*. [32] published a large systematic review evaluating serial IGRA as a potential tool to monitor treatment in patients with active TB and LTBI (30 studies; 24 used QTB-IT, three used T-SPOT.TB and three used both QTB-IT and T-SPOT.TB). No uniform pattern was seen in IGRA conversion or in reversion rates at the end of treatment for active or latent TB, and in most studies, the majority of IGRA results remained positive at the end of treatment. In the larger studies of LTBI, reversion and conversion rates were 38% and 9%, respectively, using T-SPOT.TB [21] and 24% and 18% using QTB-IT [33]. The authors concluded that although quantitative IGRA responses generally fall during treatment for TB, the large degree of variation in results among participants in each study means that IGRAs are unlikely to be useful for monitoring anti-TB treatment in clinical practice for any individual patient. In addition, we observed a large degree of variation in results of QTB-IT value between participants: the kinetics of QTB-IT showed decreased Ag IFN-γ levels both in the patients who concluded 6 months' treatment and in those who did not complete it. We also noted a decrease in Ag IFN-γ levels in three (60%) of five patients who did not receive anti-TB prophylaxis. These data were probably related to the small number of patients observed and to a great variation in previous exposure to TB before arriving at the study center. In conclusion, our study confirmed that IGRA is not reliable tool for monitoring treatment for MDR LTBI in clinical practice.

## Conflict of Interest

None declared.

## Figures and Tables

**Table 1:** Clinical characteristics of 17 subjects with positive TST and QTB-IT test for multidrug-resistant LTBI

Characteristic	Value
Male sex	17 (100%)
Age, years, mean (range)	34 (21–59)
Italian origin	4 (23.5%)
History of active TB	0
HIV, HBV, HCV infection	0
Weight, kg, mean (range)	71 (59–95)
TST induration, mm, mean (range)	20 (10–32)
Baseline QTB-IT, IU, mean (range)	18.00 (1.98–89.40)
Acceptance of LTBI treatment	12/17 (70.5%)
Conclusion of LTBI treatment	5/12 (41.6%)
Duration of LTBI treatment, days, median (range)	108 (27–180)

HBV, hepatitis B virus; HCV, hepatitis C virus; LTBI, latent tuberculosis infection; QTB-IT, QuantiFERON-TB Gold In-Tube; TST, tuberculin skin test.

**Table 2:** Characteristics of 17 subjects exposed to multidrug-resistant tuberculosis

Patient No.	Age (years)	Nationality	Duration of LTBI treatment (days)	TST induration size (mm)	Ag IFN-γ (IU/mL)		
Baseline	3 months	6 months
1	34	Nigerian	180	11.0	36.20	5.89	6.95
2	36	Italian	179	15.0	8.01	1.87	0.97
3	23	Tunisian	180	12.0	9.94	0.68	1.61
4	24	Romanian	178	25.0	3.96	4.06	1.48
5	32	Romanian	107	20.0	71.40	26.40	17.40
6	21	Tunisian	27	16.0	37.40	ND	14.30
7	27	Moroccan	79	30.0	87.00	30.50	29.80
8	43	Tunisian	70	25.0	89.40	16.40	27.90
9	36	Tunisian	94	20.0	25.00	4.05	1.71
10	36	Chinese	180	32.0	1.98	1.82	0.76
11	25	Moroccan	70	25.0	7.84	10.00	3.98
12	51	Italian	31	15.0	7.81	4.15	1.98
13	32	Nigerian	0	11.0	55.70	28.00	11.40
14	31	Moroccan	0	20.0	32.10	22.00	16.65
15	37	Moroccan	0	20.0	18.00	9.00	3.29
16	40	Italian	0	15.00	2.84	5.5	7.79
17	48	Italian	0	16.00	8.85	28.9	43.90

Ag IFN-γ, antigen-specific interferon gamma; LTBI, latent tuberculosis infection; ND, not done; TST, tuberculin skin test.

**Table 3:** AEs reported by contacts during multidrug-resistant latent tuberculosis infection treatment

AE	Patients with AEs treated with PZA/LVX, *n* (%)	Therapy discontinuation for AE, *n* (%)
Hepatitis	6 (50.0)	5 (41.6)
Diarrhoea	2 (16.6)	1 (8.3)
Gastritis	2 (16.6)	1 (8.3)
Dizziness/headache	1 (8.3)	0 (0.0)
Arthritis	1 (8.3)	0 (0.0)
None	3 (25.0)	0 (0.0)

AE, adverse event; LVX, levofloxacin; PZA, pyrazinamide.
